# Innovative therapies for IgE-mediated type I allergies 

**DOI:** 10.5414/ALX02626E

**Published:** 2026-10-30

**Authors:** Bjarke Krogstrup Jensen, Anne-Sofie Ravn Ballegaard, Josephine Baunvig Aagaard, Michaela Miehe, Edzard Spillner

**Affiliations:** Immunological Biotechnology, Department of Biological and Chemical Engineering, Aarhus University, Denmark

**Keywords:** IgE, allergy, allergen, treatment, biologics

## Abstract

Against the background of increasing prevalence of IgE-mediated type I allergies, novel approaches for treatment are urgently needed. With treatment anchored in historical developments over a century, a new layer of improvements and next-generation approaches are about to enter the stage. Advances in understanding the cellular and molecular signatures of IgE-mediated diseases, and in particular molecular technologies have paved the way for new approaches aiming at improved diagnostics and more efficient treatment. In recent years, novel technologies have been established and applied in the development of approaches which might overcome current limitations and provide novel windows of opportunity. These approaches give a first glimpse of the future treatment possibilities in allergic diseases.

## Basic principles 

Development of type I allergies is often described as a stepwise process with phases including sensitization and elicitation. Hence, treatment options fall into different categories, ranging from prevention of sensitization to blocking elicitation, modulating elicitation, and reprogramming the immune system. In addition, treatment can be based on using the allergen itself in natural or modified form or by targeting other key players of the allergic response. This review will provide an overview of novel technologies and potential future treatment options for allergic diseases. 

The current standard for treating allergy relies on symptom relievers such as anti-histamines and corticosteroids in mild-to-moderate cases, and epinephrine in more severe cases. Allergen immunotherapy (AIT) and biologics targeting regulators of diseases are mostly applied to moderate-to-severe cases of allergy (Figure 1). 

AIT is a disease-modifying treatment used for more than 100 years. Increasing doses of allergens are administered, slowly building tolerance through**, **e.g., regulatory T cells and protective blocking antibodies [1]. Variations of AIT protocols have led to different strategies including subcutaneous immunotherapy (SCIT), sublingual immunotherapy (SLIT), and oral immunotherapy (OIT) (Figure 1A), all aiming to improve effectiveness and safety [2]. Adjuvants, such as the first-generation adjuvant aluminium hydroxide (Figure 1A) currently used for SCIT in Europe, enhance the immune response and steer it towards tolerance, thereby promoting therapeutic success [3]. 

Molecular targeting of key players of the immune system has proved to be an effective alternative strategy (Figure 1B). The most prominent target is IgE, as the crosslinking of receptor bound IgE through allergens initiates the immediate response. Therefore, anti-IgE antibodies have become a mainstay since the first anti-IgE antibody omalizumab was approved for clinical use in 2003 [4]. Omalizumab captures and therefore reduces free IgE, eventually leading to downregulation of FcεRI expression and reduced activation [5]. 

Other key players in allergy include the cytokines involved in the T_H_2 immune response and their receptors. IL-4 and IL-13 both play crucial roles in the isotype switching of B cells from IgG to IgE, and IL-4 is essential for maintaining the T_H_2 phenotype [6]. Although direct anti-IL-4 concepts failed, the anti-IL-4Rα antibody dupilumab (Figure 1B), preventing binding of both IL-4 and IL-13, was approved for use in atopic dermatitis (AD) [7]. Tralokinumab and lebrikizumab target IL-13 directly and have also been approved for use in AD [8, 9]. Another important cytokine is IL-5, modulating the contribution of eosinophils to the allergic response [6]. Both the anti-IL-5 antibody mepolizumab and the glycoengineered anti-IL-5Rα antibody benralizumab are approved for treatment of severe eosinophilic asthma (Figure 1B) [10, 11]. 

## Cutting edge 

The standard treatment of type I allergy in the form of AIT is lengthy, comes with risk of adverse events, and has limited treatment adherence, while treatment with biologics remains expensive. Therefore, novel treatment strategies are needed. For AIT, new routes of administration are being explored (Figure 1A), such as intralymphatic immunotherapy (ILIT) which aims at reducing the dose and number of injections by direct administration into the lymph nodes [12, 13]. In addition, epicutaneous (EPIT) and liver-targeted immunotherapy (LIT) utilize the Langerhans cells in the cutaneous immune system and the hepatic antigen-presenting cells in the liver, respectively, to promote a regulatory T-cell response and thereby tolerance [14, 15]. The content of AIT treatment is shifting from the use of allergen extracts with simple first-generation adjuvants to the use of second-generation adjuvants (Figure 1A) such as the toll-like receptor (TLR) ligands: monophosphoryl lipid A (MPLA), flagellin, and CpG oligodeoxynucleotides. These induce a T_H_1 response with MPLA and CpG oligodeoxynucleotides, additionally inducing IgG production and CD8^+^ T-cell response, respectively, promoting therapeutic success [3, 16]. Another area of second-generation adjuvants includes biodegradable nanoparticles such as liposomes, polyesters, virus-like particles (VLP), and more. These are being utilized as carriers of allergens and adjuvants, either on the surface or inside the nanoparticle, steering the immune response in a T_H_1-biased direction [17]. Various VLPs are already approved for clinical use in vaccines where they have shown great immune-modulating capabilities and would therefore be highly suitable for AIT [18]. Lipid nanoparticles are also being utilized in mRNA vaccines which quickly gained ground under the SARS-CoV-2 pandemic. This strategy is also applied to AIT using mRNA encoding the major allergens. A study on house dust mite allergy in mice treated with allergen-encoding mRNA encapsulated in a lipid nanoparticle suppressed T_H_2-type inflammation, induced a T_H_1 response, and induced protective IgG production [19]. Plant-based enveloped bioparticles displaying Ara h 2 or Der p 2 have also shown promising results, being hypoallergenic and promoting an IgG mediated response [20, 21]. In combination with second-generation adjuvants, recombinant proteins are being explored in AIT in an increasing manner as they become more readily available. Hypoallergenic variants of allergens have been generated through genetically engineering and chemically modifying recombinant allergens [22, 23, 24]. To maintain high stability of hypoallergenic allergens, a new strategy of encapsulating them in protein bodies might protect them from proteolytic digestion in the gastrointestinal tract and therefore allow for their use in OIT [25]. 

In contrast to these approaches, molecular targeting as a therapeutic strategy is also evolving, with new technologies and novel targets paving the way (Figure 1B). Direct targeting of the relevant allergens is realized in passive immunotherapy, with protective blocking antibodies administered to the patient. Proof-of-concept has been established for cat, birch, and peanut allergy using antibody cocktails [26, 27, 28]. More recently, passive immunotherapy against birch and honeybee venom allergen using single, multimeric or multiepitopic nanobody-based formats has been demonstrated [29, 30]. Such advanced antibody formats demonstrate superior blocking and protection against anaphylaxis. Blocking activity is also realized by mimotopes, epitope-mimicking peptides, which bind to specific IgE. This concept is currently being explored, e.g., with Ara h 2 mimotopes expressed on *Lactococcus lactis* as carrier [31]. 

Targeting of the key molecule IgE with omalizumab has been used as stand-alone treatment and combined with AIT as a pre-treatment to capture free IgE before introducing the allergens (Figure 1A) [32]. This has shown to reduce the risk of adverse events without impacting the development of protection [33]. Novel anti-IgE antibodies are also being explored (Figure 1B), such as UB-221 which binds an epitope separate from the low affinity receptor CD23 (Figure 2A) allowing binding of IgE:UB-221 complexes to CD23, promoting CD23-mediated downregulation of IgE [34]. 

A more recent strategy in anti-IgE antibodies relies on disruption of the IgE:FcεRI complex, as this would allow desensitization of cells. Antibodies including HMK-12, omalizumab variants, and the single domain antibody, or nanobody 026 have all shown the ability to displace IgE from the FcεRI (Figure 2B) [35, 36, 37]. The omalizumab variant C03 with a flexible linker shows higher affinity and disruptive capabilities compared to omalizumab, highlighting the importance of affinity and flexibility in molecular targeting [38]. 

As a variation of anti-IgE approaches, an IgE vaccine has also been generated consisting of the Cε3-4 domain of human IgE coupled to a carrier protein. Immunization showed efficacy in a humanized mouse model with anti-hIgE antibodies still detectable after 12 months [39]. At last, directly targeting the FcεRI using a monovalent anti-FcεRIα antibody avoiding cross-linking has shown protection in mice [40]. 

Other novel targets include the immunoregulatory receptor sialic acid-binding immunoglobulin-like lectin (siglec) 6 expressed on mast cells. Engagement with the anti-siglec-6 antibody AK04 (Figure 1B) in mice showed inhibition of anaphylaxis [41]. Another target on mast cells is the stem cell factor receptor KIT which is responsible for mast cell development, proliferation, maturation, and overall survival. Therefore, mast cell depletion as treatment of chronic spontaneous urticaria can be realized using anti-KIT receptor antibodies such as briquilimab and barzolvolimab which are currently in phase 1/2 and 3 clinical trials, respectively [42]. The OX40 receptor is expressed by activated skin-homing T cells and plays a role in AD, as it is responsible for the activation and formation of memory T cells [43]. Targeting with the anti-OX40 receptor antibody rocatinlimab has been examined in a phase 3 clinical trial showing significant clinical improvements while being well tolerated [44]. 

Multispecific targeting is being used to agonize cytokines with higher affinity and increased blocking effect. A rather easy generation of multimeric antibodies is achieved by the use of nanobodies due to their nature as single domain binders found in camelid species. Nanobodies can be combined in, e.g., sequential or multichain formats. Their small size also improves tissue penetration without compromising specificity due to their often longer than conventional complementarity-determining region 3, which additionally enables targeting of concave epitopes such as active sites [45, 46]. As an example, a nanobody trimer targeting IL-13, which drives eosinophilic inflammation, showed high avidity and IL-13-blocking efficacy and could therefore be beneficial [47]. Another strategy using multivalent antibodies is to agonize multiple cytokines simultaneously. One such example is lunsekimig (Figure 1B) consisting of 5 nanobodies fused by amino acid linkers. Two of these nanobodies target TSLP which initiates asthma inflammatory cascades, two target IL-13, and one targets albumin for prolonged half-life. The phase 1 trial was successful and showed a decrease in asthma-related endpoints [48]. Multispecific targeting is also being used to prevent the uptake of airway allergens by epithelial cells through bispecific targeting of the allergen and intracellular adhesion molecule (ICAM)-1 (Figure 1B). In this approach, ICAM-1 is used as an anchor on epithelial cells, allowing trapping of the allergens entering the airways. This was realized in an antibody combination consisting of anti-ICAM-1 and anti-Phl p 2 and Phl p 5 antibodies [49]. 

## Conclusion 

The current standards of type I allergy treatment are expensive and lengthy, have a suboptimal compliance, and come with a risk of adverse events. However, AIT is currently being improved through novel delivery routes, chemically or biologically engineered allergens, and the use of second-generation adjuvants, promising lower doses, fewer injections, and less side effects. 

Molecular targeting of key players is also evolving with novel targets and concepts and might allow for precise and personalized treatment in the future. Another aspect being explored is the targeting molecule itself. Multispecificity is increasingly utilized to improve avidity and inhibit multiple targets at the same time, resulting in more substantial inhibition of the allergic response. 

The improvement of treatment will eventually benefit the clinicians as treatment becomes less labor-intensive and more personalized. And as the number of molecular tools, novel targets, and new treatment concepts emerge, the benefits will only increase for both clinicians and patients. 

## Authors’ contributions 

B.K.J. wrote the first draft, all authors provided further scientific guidance, editing and proof-reading. Figures 1 and 2 were prepared by B.K.J. and E.S. 

## Funding 

E.S. obtained funding from the Aarhus University Research Foundation, AUFF-E-2022-9-44. 

## Conflict of interest 

The authors declare to have no conflict of interest in relation to this review. 

**Figure 1:**
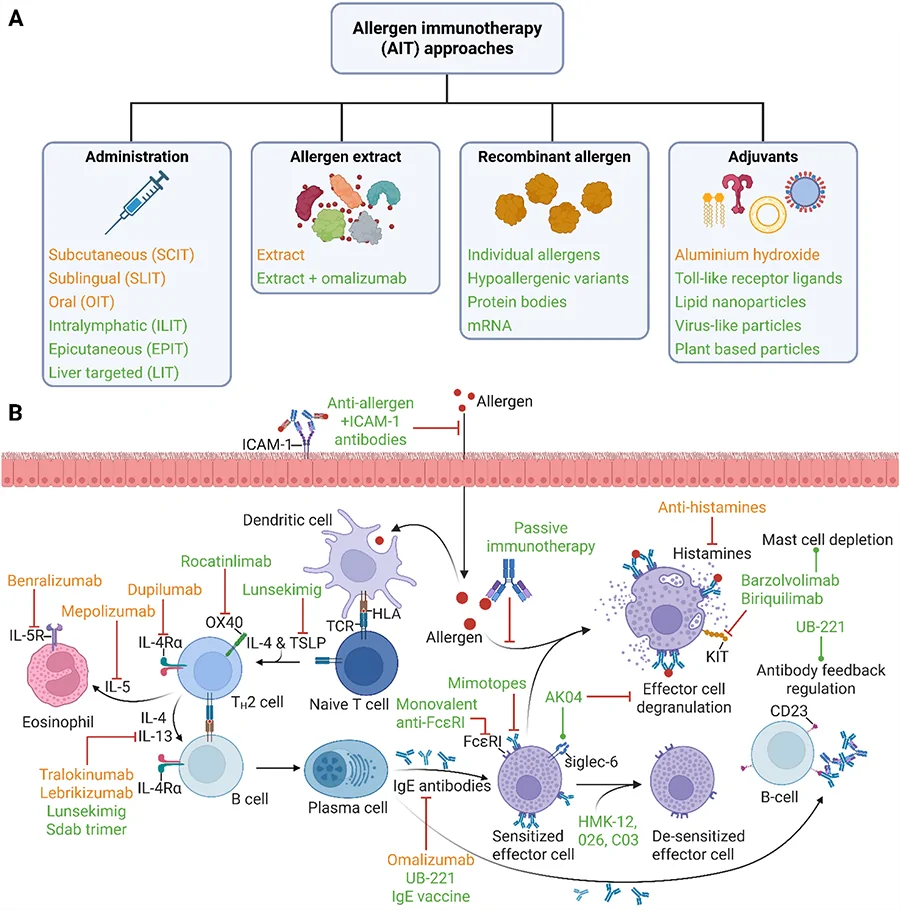
Overview of type I allergy treatment strategies with A) highlighting approaches within allergen immunotherapy and B) displaying sensitization and elicitation with molecular targets and therapeutics. Green: cutting edge, orange: established concepts, red line: blocking effect, green line: inducing effect. Allergen immunotherapy (AIT), T-cell receptor (TCR), human leukocyte antigen (HLA), thymic stromal lymphopoietin (TSLP), intracellular adhesion molecule (ICAM), Janus kinase (JAK), sialic acid-binding immunoglobulin-type lectin (siglec). Created in BioRender. Spillner, E. (2026) https://BioRender.com/lh6gqgd.

**Figure 2:**
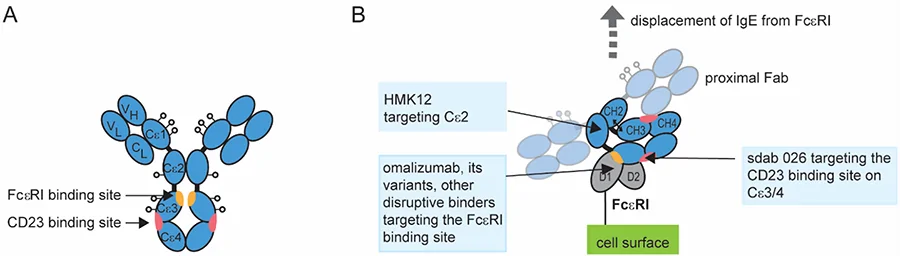
A: Illustration of IgE with FcεRI binding site highlighted in yellow and CD23 binding site highlighted in red. B: Illustration of IgE bound to the FcεRI receptor (grey) with binding sites of disruptive anti-IgE therapeutics shown with arrows.
